# Determinants of Medical Help–Seeking Behavior Following Case Finding of Early Cognitive Impairment: Semistructured Interview Study of Patients and Caregivers

**DOI:** 10.2196/79386

**Published:** 2026-05-19

**Authors:** Shu Rong Lim, Xiaohui Xin, Gerald Choon Huat Koh, Julian Thumboo, Tau Ming Liew

**Affiliations:** 1 Health Services Research Unit, Singapore General Hospital, Outram Road, Singapore, Singapore; 2 Saw Swee Hock School of Public Health, National University of Singapore, Singapore, Singapore; 3 Department of Rheumatology and Immunology, Singapore General Hospital, Singapore, Singapore; 4 SingHealth Duke-NUS Medicine Academic Clinical Programme, Duke-NUS Medical School, Singapore, Singapore; 5 Department of Psychiatry, Singapore General Hospital, Outram RoadSingapore, 169608, Singapore, 65 62223322; 6 Health Services Research and Population Health, Duke-NUS Medical School, Singapore, Singapore

**Keywords:** behaviour change wheel, COM-B, dementia, framework analysis, medical help-seeking, mild cognitive impairment, qualitative research methods

## Abstract

**Background:**

Timely medical follow-up after a diagnosis of cognitive impairment, such as mild cognitive impairment (MCI) or dementia, is imperative for initiating appropriate medical treatment and accessing comprehensive care management and psychosocial support. However, many community-dwelling older adults who receive a positive case-finding result default on their medical follow-up appointments. This persistent challenge undermines early detection and active case-finding efforts and increases the risk of early institutionalization. Understanding the determinants is important for developing effective interventions in community-based case-finding.

**Objective:**

This qualitative study aimed to explore the barriers and facilitators influencing medical help–seeking behavior among community-dwelling individuals in Singapore diagnosed with MCI or dementia following a positive case-finding result. The COM-B (capability, opportunity, motivation-behavior) framework of the Behavior Change Wheel (BCW) was used to systematically identify these determinants.

**Methods:**

Using maximum variation sampling based on age, gender, ethnicity, living arrangement, relationship with caregiver, prior memory concerns, and whether participants had sought medical follow-up after their cognitive impairment diagnosis, we conducted 21 unique household semistructured interviews with 26 individuals (comprising 5 participant-caregiver dyads of MCI, 6 participants diagnosed with MCI, 1 caregiver of a participant with MCI, and 9 caregivers of participants diagnosed with dementia). These participants were a subset of individuals and caregivers recruited from a community-based study validating an artificial intelligence (AI)–based dementia case-finding tool. Interviews were audio-recorded, transcribed verbatim, and analyzed using the framework analysis method. Two authors (SRL and XX) performed inductive coding before mapping to COM-B, with subsequent discussion and review by the remaining authors. The COM-B components informed the selection of the relevant intervention functions from the BCW.

**Results:**

Barriers to medical help–seeking behavior after cognitive impairment diagnosis included physical impairments, low health literacy to navigate the health care processes, inaccurate disease knowledge, complicated health care processes, inadequate physical infrastructure to navigate health care organizations, oversimplified cultural representation of dementia, perceived inaccuracy of the case-finding tool, and lay beliefs about seeking medical care when sick. Facilitators included adopting strategies to track medical appointments, a case-finding result letter as a cue for action, caregivers with profamily workplace policies, and valuing proactive medical disease management. Notably, social capital, (in)ability to recognize symptoms, and strong affective states triggered by the case-finding results were both barriers and enablers.

**Conclusions:**

These findings highlight a complex interplay of individual, social, and structural determinants influencing medical help–seeking behavior following a cognitive impairment diagnosis. Rather than a linear trajectory, medical help–seeking is shaped by a dynamic interplay across the COM-B domains. Drawing on these findings, we developed a set of comprehensive and actionable recommendations grounded in the BCW intervention functions to support timely medical follow-up after a cognitive impairment diagnosis. To maximize their impact, these recommendations should be collaboratively refined and evaluated with stakeholders using the acceptability, practicability, effectiveness, affordability, side effects, and equity (APEASE) criteria.

## Introduction

### Background

The global prevalence of dementia is projected to increase substantially, from 57.4 million cases in 2019 to 152.8 million in 2050, primarily due to population growth and an aging population, with a greater proportion of cases occurring among women than men [1]. International bodies such as Alzheimer’s Disease International [2] and the International Association of Gerontology and Geriatrics [3] have advocated for active case-finding of dementia among high-risk older adults. This involves extending beyond tertiary hospitals to proactively identify high-risk individuals through routine annual cognitive evaluations in community-dwelling older adults [3]. However, the potential benefits of these cognitive evaluations in the community are diminished when individuals do not pursue timely medical follow-up after receiving a positive case-finding result for cognitive impairment (ie, mild cognitive impairment [MCI] or dementia). Timely medical follow-up is essential, not only for initiating appropriate medical treatments (eg, cognitive enhancers), but also for accessing comprehensive care management (eg, behavioral, financial, legal, and end-of-life issues), and social support (eg, support for caregivers) [4]. Delayed follow-up can have far-reaching consequences, including compromised well-being [5,6] and increased risk of early institutionalization in long-term care facilities [7-9].

Despite these risks, little is known about the frequency of actual medical help–seeking following a diagnosis of cognitive impairment. A large national survey conducted in China between 2015 and 2018 revealed that 71.4% (1974/2766) of individuals with dementia and 97.2% (6926/7125) of individuals with MCI did not seek medical treatment [10]. In Singapore, unpublished data from a pilot case-finding study [11] by the corresponding author (TML) as part of a larger implementation project to train and implement PENSIEVE-AI, an artificial intelligence (AI) tool for early cognitive impairment detection in the community, further illustrate this challenge. Among community-dwelling older adults diagnosed with cognitive impairment and referred directly to the Singapore General Hospital (SGH) memory clinics, 20.8% (10/48) defaulted on their appointments. Of these individuals, 8 were diagnosed with MCI and 2 with dementia. These missed follow-ups occurred despite deliberate efforts to facilitate medical follow-up, including direct referrals to SGH memory clinics for individuals living near SGH, text message appointment reminders, and proactive coordination by a clinic case manager before follow-up appointments. Although these data were derived from a single, relatively well-resourced acute tertiary hospital in Singapore, they likely underestimate the extent of the default follow-up rates in the broader population. Together, these findings highlight a persistent and multifaceted challenge of timely medical follow-up after receiving a diagnosis of cognitive impairment. Addressing this gap requires a better understanding of the determinants of medical help–seeking behavior in this context.

### Existing Evidence on Medical Help–Seeking Determinants

A systematic review [12] identified 5 key illness perception factors associated with delayed help-seeking among individuals with dementia and their caregivers, including cultural beliefs of caregiving duties, acceptance of diagnosis, lack of knowledge about dementia, complex health care systems (including both the positive and negative experiences from prior health care professionals and services), and perceived threat to personal independence. However, the review focused on help-seeking intentions rather than actual help-seeking behaviors. This distinction is important due to the well-established intention-action or intention-behavior gap in health behaviors [13-15]. For clarity, throughout this paper, help-seeking without the qualifier medical refers to both formal (ie, health care professionals) and informal (ie, family and friends) sources of help-seeking support. In contrast, medical help–seeking behavior refers specifically to the actual actions taken to seek and obtain care from formal health care providers, such as neurologists, psychiatrists, or general practitioners.

Building on this, a recent qualitative study [16] extended medical help–seeking intentions in individuals with dementia and identified 8 key determinants of medical help–seeking behavior among individuals with MCI. These determinants can be conceptually clustered into 3 broad categories, including psychological processes, comprising disease knowledge, perceived disease threat, symptom attribution, negative valence emotions, and reliance on cognitive strategies; socioeconomic and structural conditions, that is, financial stability and accessible health care services; and social support. However, participants in this study were recruited from a memory clinic and were already on medical follow-up. As such, the determinants influencing individuals with MCI who did not seek medical help remain underexplored, leaving an important unaddressed gap in the context of medical help–seeking following positive case-finding in the community.

### Study Aim

To address these methodological and practical knowledge gaps, this qualitative study aims to explore the barriers and facilitators influencing medical help–seeking behavior among individuals diagnosed with MCI or dementia following positive community-based case-finding results in Singapore. Specifically, what are the barriers and facilitators to medical help–seeking behavior among community-dwelling individuals diagnosed with cognitive impairment following a positive case-finding result? This study forms part of a larger implementation effort involving the deployment of PENSIEVE-AI but is methodologically independent from the PENSIEVE-AI tool development study [11]. In other words, although the participants in this qualitative study were recruited from the PENSIEVE-AI pilot study [11], this study focuses specifically on exploring the determinants of medical help–seeking after MCI or dementia diagnosis. Given the complex, multifactorial nature of medical help–seeking behavior, a theory-informed approach was adopted to guide data collection and analysis. This approach enables the systematic identification of key determinants grounded in an established theoretical framework to inform the future development of targeted interventions and implementation strategies to improve medical help–seeking follow-up [17].

### Theoretical Framework

This study applies the capability, opportunity, motivation-behavior (COM-B) framework [18,19], a widely used systematic framework for understanding behavior, designing targeted interventions, and achieving health behavior change across diverse settings, including dementia screening [20]. Within this framework, capability refers to the physical and psychological ability to perform the behavior; opportunity pertains to the physical and social environments; and motivation encompasses both automatic processes, such as emotions and impulses, and reflective processes, such as evaluation and plans [18,19]. Its utility lies not only in identifying behavioral determinants but also in linking the COM-B diagnosis with the 9 intervention functions that encircle the COM-B framework of the behavior change wheel (BCW): education, persuasion, enablement, incentivization, coercion, training, restrictions, environmental restructuring, and modeling [18,19,21]. This allows for the systematic development of tailored strategies specific to the behavioral determinants [18,19].

## Methods

### Study Setting

This study arose from a recent pilot case-finding study in Singapore that screened approximately 1800 community-dwelling older adults for early cognitive impairment using the PENSIEVE-AI tool [11]. Multimedia Appendix 1 outlines the steps of the PENSIEVE-AI case-finding pilot study. Community-dwelling older adults were recruited and underwent comprehensive cognitive assessments. Assessments of MCI, dementia, or normal cognition were made by 3 dementia specialists using an internationally recognized gold-standard consensus approach based on all the information from the comprehensive assessment. Community-dwelling older adults living in the SGH catchment area with positive case-finding results for MCI or dementia received direct medical appointments for follow-up at SGH memory clinics, whereas those living outside the SGH catchment area were strongly advised to seek medical attention from primary care doctors for referral to memory clinics within their home vicinity.

### Study Design and Participant Selection

**Figure 1.**
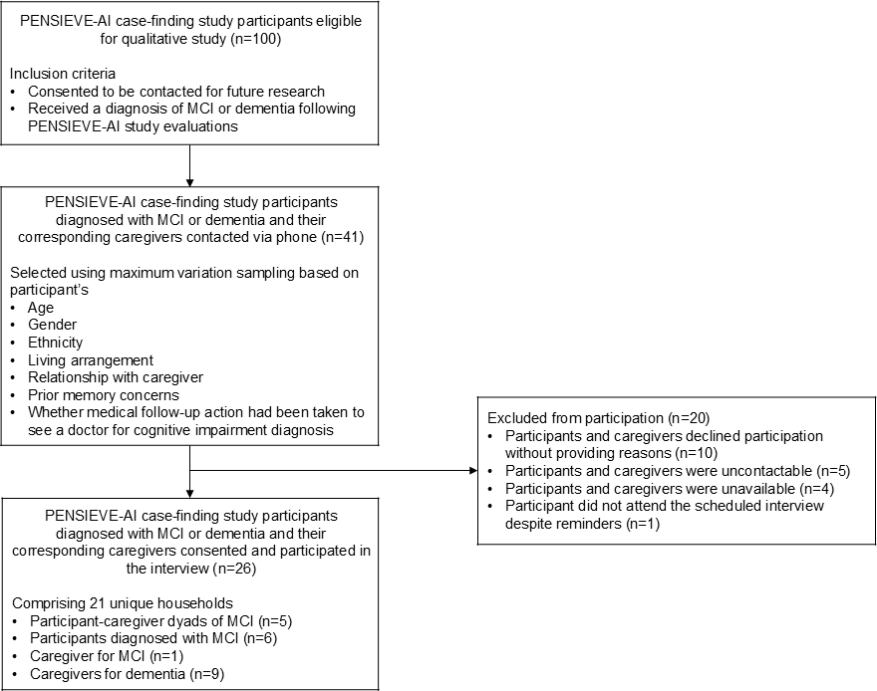
Qualitative study recruitment and selection flowchart for participants and caregivers. MCI: mild cognitive impairment.

Forty-one eligible PENSIEVE-AI participants diagnosed with MCI or dementia and their corresponding caregivers from the pilot case-finding study [11] were contacted for the semistructured interviews over the phone. Participants who declined to participate did not preclude their caregivers from participating in the study. Interviewees were selected using maximum variation sampling to capture diverse experiences of medical help–seeking following positive case-finding. Sampling variation was based on participants’ age, gender, ethnicity, living arrangement, relationship with caregiver, prior memory concerns, and whether participants had sought medical follow-up after their PENSIEVE cognitive impairment diagnosis. These characteristics were selected because they are expected to influence the cognitive impairment appraisal, access to formal and informal support for medical help–seeking, and caregiver involvement in decision-making regarding medical help–seeking. Figure 1 details the recruitment and selection process.

For participants diagnosed with dementia, only their caregivers were interviewed due to practical considerations such as impaired decision-making capacity, reliance on interviewer’s skill for consistent self-reports, and risk of distress during lengthy interviews. For participants diagnosed with MCI, interviews were conducted with participants alone, with participants and caregivers together, or with caregivers only, depending on participant preferences. This strategy aimed to improve interview participation rates. Interviews were conducted in either English or Mandarin Chinese. All interviews were audio recorded, anonymized, transcribed verbatim, and verified by another author (SRL or XX). Mandarin Chinese interviews were transcribed verbatim in the source language, coded in English, and only the extracts were translated into English by SRL and verified by XX. All authors are bilingual and proficient in English and Mandarin. All interviews were conducted by SRL, with XX accompanying one interview when a male participant was home alone. Interviews continued until data saturation was reached. An interview guide based on COM-B [18] was developed and used for the semistructured interviews (Multimedia Appendix 2). The COREQ (Consolidated Criteria for Reporting Qualitative Research) checklist was used as a guide for reporting our study [22]. Informed consent was taken at the start of the interview.

### Data Analysis

The data were analyzed using framework analysis [23] involving the following steps: (1) familiarizing with the interviews and transcripts; (2) independent inductive coding of the transcripts (ie, open coding) using Microsoft Word’s comments function and the coding macro [24] to export the codes and the associated text to Microsoft Excel; (3) grouping codes into defined facilitator and barrier categories; (4) indexing the transcripts according to the existing codes and categories; (5) charting the categories into the COM-B matrix, including quotation references; and (6) performing data interpretation.

The identified COM-B components were used to guide the selection of relevant intervention functions from the BCW. The coding, indexing, charting, and selection were performed by SRL and XX, who have respective backgrounds in psychology and sociology. The categories were developed together through discussion with TML, who has expertise in geriatric psychiatry and public health, and subsequently reviewed by the remaining authors with expertise in geriatric medicine, medicine, health services research, and public health. Disagreements in the categorization of barriers and facilitators, mapping to COM-B components, and selection of intervention functions were resolved through discussion. We maintained an audit trail of coding comments, categorization and selection decisions, and engaged in reflexive discussions throughout the analysis.

### Ethical Consideration

This study was approved by the SingHealth Centralized Institutional Review Board (reference number 2021/2590). Informed consent procedures for participants with cognitive impairment included a simplified capacity assessment in accordance with the Mental Capacity Act. Participants were required to demonstrate their ability to understand, retain, and use the study information to make an informed decision, and to communicate their decision to participate by paraphrasing key information, including the voluntary nature of participation and the right to withdraw at any time. The interviewer remained attentive to verbal and nonverbal cues of distress and paused interviews when necessary to ensure participants’ and caregivers’ well-being and to accommodate caregiving responsibilities. Participants and caregivers were reminded that they could terminate the interview at any time, and breaks were offered when needed. Data were kept confidential, with access restricted to the study team. Interview data were anonymized during transcription, with identifiable information removed and pseudonyms assigned where appropriate. Participants received Singapore $40 (US $1=1.26 Singapore Dollars) as a token of appreciation.

## Results

### Overview

Between May 2023 and January 2024, 26 individuals were interviewed across 21 unique households, comprising 12 households with a family member diagnosed with MCI and 9 households with a family member diagnosed with dementia. The mean interview duration was 53 minutes, ranging from 25 to 71 minutes. Of the 21 household interviews, 17 were conducted in person, and 4 by telephone. The median age group for participants with MCI was 70‐79 years, while the median age group for the interviewed caregivers was 60‐69 years. Most interviewees were Chinese, living with family members in a 4‐5 room public housing apartment. Notably, 23.8% of households did not take further medical follow-up action after the positive findings from the pilot case-finding program, with 25.0% for MCI households and 22.2% for dementia households. Detailed sociodemographic characteristics of the interviewees are presented in Table 1. Five interviews were conducted in Mandarin Chinese.

**Table 1.** Sociodemographic characteristics of participants diagnosed with mild cognitive impairment (MCI) and caregivers of participants with MCI or dementia (n=26, across 21 unique household interviews: 5 participant-caregiver dyads of MCI, 6 participants diagnosed with MCI, 1 caregiver of a participant with MCI, and 9 caregivers of participants diagnosed with dementia).

Characteristic^a^	Participants with MCI^b^ n=11	Caregivers of participants with MCI n=6	Caregivers of participants with dementia n=9
Age group (years)			
30‐39	—^c^	1	—
50‐59	—	1	4
60‐69	4	3	1
70‐79	4	1	3
80‐89	3	—	1
Sex			
Female	6	4	8
Male	5	2	1
Ethnicity			
Chinese	9	4	8
Malay	—	1	—
Indian	1	1	1
Eurasian	1	—	—
Marital status			
Widowed/divorced/separated	3	1	—
Married	5	4	5
Single	3	1	4
Type of housing			
1‐2 room public housing apartment	3	2	—
3-room public housing apartment	2	—	1
4‐5 room public housing apartment	5	4	4
Private apartment/condominium	1	—	3
Landed property	—	—	1
Work status			
Retired/unemployed	9	2	6
Working	2	4	3
Living arrangement			
Lives alone	3	1	—
Lives with family	8	5	9
Attended medical follow-up for cognitive impairment			
Yes	8	1^d^	7^e^
No	3	—	2^e^
Type of caregiver’s relationship with the participant			
Spouse	—	2	2
Child	—	3	5
Sibling	—	—	2
Neighbor	—	1	—
Interview setting			
Home interview	4	1	2
Meeting room interview on the hospital campus	6	3	5
Phone interview	1	2	2

a Interviewees comprised 5 participant-caregiver dyads of MCI, 6 participants diagnosed with MCI, 1 caregiver of a participant with MCI, and 9 caregivers of participants with dementia.

b MCI: mild cognitive impairment.

c Not applicable.

d Denotes a participant who was not interviewed due to a language barrier.

e Denotes participants with dementia who were not interviewed.

**Figure 2.**
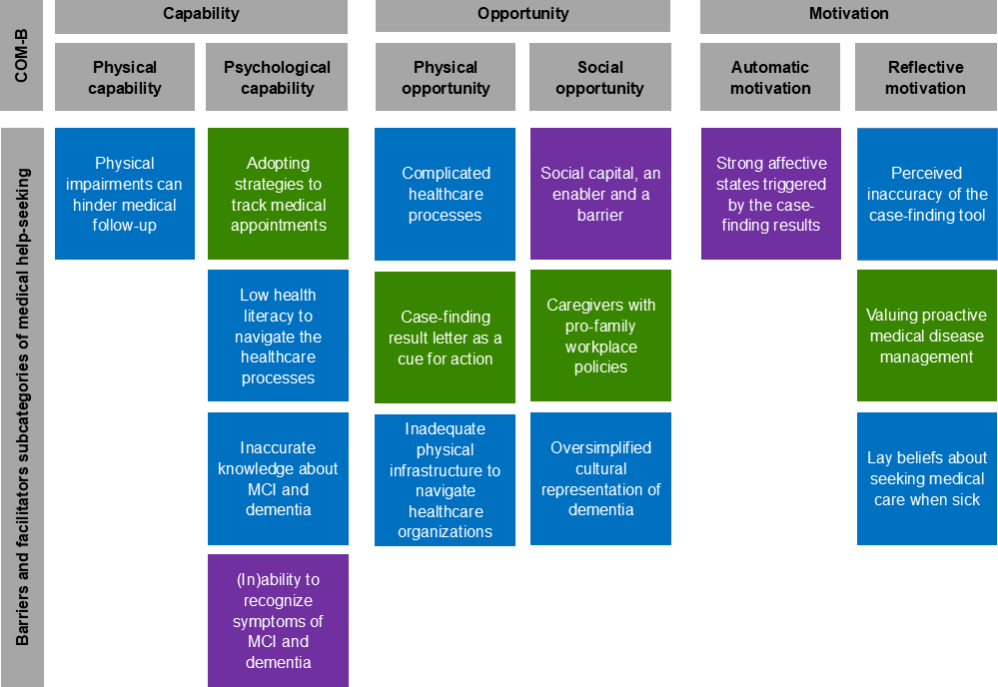
Determinants of medical help–seeking behavior following mild cognitive impairment (MCI) or dementia diagnosis, mapped onto the capability, opportunity, motivation-behavior (COM-B) domains and subdomains. Blue-colored boxes indicate barriers; green-colored boxes indicate facilitators; and purple-colored boxes indicate determinants that are both barriers and facilitators. COM-B: capability, opportunity, motivation-behavior; MCI: mild cognitive impairment.

Based on the in-depth interviews, we identified 15 determinants encompassing both facilitators and barriers of medical help–seeking behavior. These determinants were mapped onto the COM-B domains. Detailed descriptions of each determinant, along with illustrative verbatim quotes, are detailed in the sections below. Figure 2 provides an overview of these determinants as mapped onto the COM-B domains and subdomains.

### Capability

#### Physical Impairments Can Hinder Medical Follow-Up

Two types of physical impairment were identified as barriers to timely medical follow-up after cognitive impairment case-finding results. First, mobility limitations necessitated reliance on caregivers for transportation, making the decision to attend medical follow-up appointments not solely the participant’s, but also contingent on the availability of social support (Extract 1).

Extract 1

*… she can’t walk very well … so most of the time my husband … will drive her. So I have to also cater to my [husband’s availability too]- sometimes my husband cannot make it.*

[Caregiver 8 of participant with dementia]

Second, speech and hand impairments, particularly among stroke survivors, can impede participants’ ability to independently manage appointment scheduling by phone or text messages (Extract 2).

Extract 2

*Speaking to a person sometime[s] may have a problem … (In dialect) one duck one chicken … you communicate they can’t get you. Sometimes over the phone … either I cannot … Because I have (sic) stroke … now I still can talk la but the slur is still there … [When typing on the phone,] it goes to the next alpha numeral … [as] I can’t type with this [disabled] hand …*

[Participant 5 with MCI]

#### Adopting Strategies to Track Medical Appointments

A notable facilitator of psychological capability was the adoption of practical strategies to track medical appointments. To mitigate forgetfulness and minimize missed medical appointments, participants and caregivers used resourceful strategies, such as diligently recording appointment dates on physical or digital calendars (Extracts 3 and 4) and prominently displaying appointment letters in a conspicuous part of their living spaces.

Extract 3

*… when [I have an] appointment … I must note it immediately [on the physical calendar]. If I delay, I will tend to forget …*

[Participant 1 with MCI]

Extract 4

*… when I have the [doctor’s] appointment … I surely will keep it up … I will put it down [in my phone calendar] …*

[Participant 2 with MCI]

#### Low Health Literacy to Navigate the Health Care Processes

Low health literacy, defined as the limitation in the degree to which individuals have the ability to find, understand, and use information and services to make informed health-related decisions and actions for themselves or others [25], posed a barrier to timely follow-up after case-finding of cognitive impairment. Participants and caregivers, who lacked the familiarity with, and necessary skills to navigate, the health care processes (eg, rescheduling medical appointments to follow up on cognitive impairment case-finding results) had to rely on family members living elsewhere for assistance, thereby causing further delays or defaults on their medical appointments (Extract 5).

Extract 5

*No, I don’t know [we can bring forward medical appointments]. My appointments also I don’t know. My sister [who does not live with me] will do [it] for me … when she comes [by to visit us] in the evening.*

[Caregiver 9 of participant with dementia]

#### Inaccurate Knowledge About MCI and Dementia

Three types of inaccurate knowledge about cognitive impairment were identified: (1) misperceiving symptoms as normal aging, (2) misinterpreting cognitive function improvement, and (3) misunderstanding disease progression. First, participants and caregivers’ understanding of MCI and dementia was primarily based on traditional lay beliefs rather than scientific knowledge, rooted in the common misperception that the symptoms of MCI and early dementia were a normal part of aging rather than a medical condition (Extract 6). As a result, these symptoms were not considered sufficient to warrant medical attention.

Extract 6

*Caregiver: He just said he’s 85 [years old], he’s just forgetful …*

*Participant: I feel that I’m just being forgetful only [due to my age]*

[Caregiver 7 and participant 7 with MCI]

Second, participants and their caregivers described perceived subjective improvement in participants’ cognition following treatment for other health conditions, which contributed to delays or defaults in seeking medical follow-up on case-finding results (Extract 7).

Extract 7

*It is nothing unusual for him [to have MCI] as he’s over 70 years already … you will definitely have it (MCI) when you are old … it is just a matter of degree … there is no need to see a doctor for it … because he’s receiving regular vitamin [B12] injection at the polyclinic, I feel that his recent memory is better, more stable … there’s [an] improvement [in his memory].*

[Caregiver 10 of participant with MCI]

The third inaccuracy in the lay understanding of cognitive impairment arises from a life course perspective of the disease trajectory. While participants and their caregivers recognized the value of medical care to delay disease progression, they did not anticipate the cognitive impairment progressing to the disabling state of severe dementia within the participants’ remaining lifetime. They anticipated other health issues to take precedence, diminishing the perceived benefit in seeking medical treatment for cognitive impairment (Extract 8).

Extract 8

*… so after all, she’s 83. You don’t know, maybe before she reached that [advanced] stage [of dementia], she has (sic) some [other] diseases.*

[Caregiver 16 of participant with dementia]

#### (In)ability to Recognize Symptoms of MCI and Dementia

The ability to recognize the signs and symptoms of MCI and early dementia as abnormal was a key facilitator in legitimizing the need to prompt participants to seek further medical attention (Extract 9).

Extract 9

*… [I] left the stove on and I went out … I may lock the door, but I will leave my key … at the gate … there were a number of times [I did that] … Then the other thing … was that I [am] not certain how to pronounce this word … I don't think [it] is normal. That was the trigger [to seek medical attention for MCI] … that was my motivation.*

[Participant 1 with MCI]

Caregivers who recognized these signs used the positive case-finding result to confirm their suspicions and used the diagnosis to encourage resistant family members who disagreed about the nature of their symptoms to pursue medical follow-up. However, denial remained a barrier despite these efforts (Extract 10).

Extract 10

*… [the PENSIEVE-AI tool result] was expected … it’s just to confirm the diagnosis and then at the same time hopefully can make her wake up and acknowledge that she has this issue. But she is still in denial. Up to today she still says … it’s normal aging … old folks around her age are like that so she doesn’t think it’s an issue.*-

[Caregiver 16 of participant with dementia]

In other cases, both the participants and the caregivers failed to recognize the symptoms of cognitive impairment and dismissed them, leading to inaction, which constitutes a major barrier (Extract 11).

Extract 11

*… there’s no signs like he’s getting worse off or he’s getting bad … he also thinks that it’s nothing wrong … it’s basically more like probably because he’s not occupied …*

[Caregiver 6 of participant with MCI]

### Opportunities

#### Complicated Health Care Processes

Three barriers within the health care processes impeded timely medical follow-up on cognitive impairment case-finding results. The first was variability in the referral process. Participants living near SGH received direct appointments arranged by a hospital coordinator to follow up on the case-finding results with a specialist at the SGH memory clinic (Extract 12).

Extract 12

*This was an appointment [date] given by [the hospital]. [If the hospital did not provide an appointment date], we will not go [to see the doctor]. We will wait for the doctor, [the doctor] can message me if they have something to tell me.*

[Caregiver 3 of participant with MCI]

In contrast, those living further away were advised to obtain a referral from a primary care doctor to see a memory clinic specialist by themselves. This process introduced at least 2 potential risks for appointment delays and defaults: oversight by the participants, their caregivers, or both due to other commitments (Extract 13), and potential dismissal of the referral letter by primary care doctors (Extract 14).

Extract 13

*… I [am] also forgetful … even though I went to the polyclinic with her [recently for urine infection], I forgot to ask the doctor for a referral [to see a specialist for dementia].*

[Caregiver 8 of participant with dementia]

Extract 14

*But because the doctor is the one that told him there is no need to come … so he never see[s] that doctor again …*

[Caregiver 6 of participant with MCI]

The second barrier was the perceived high cost of laboratory investigations (eg, blood tests, brain imaging; Extract 15) that deterred medical follow-up.

Extract 15

*The issue … [for] her is [the brain imaging scan is] going to cost me a lot of money.*

[Caregiver 3 of participant with MCI]

Some participants and their caregivers were reluctant to make out-of-pocket cash payments for further investigations, with some preferring to use funds from their MediSave accounts, Singapore’s national medical savings scheme. The MediSave annual Singapore $300 (US $236) withdrawal limit for outpatient diagnostic and treatment imaging per person influenced decisions to medical follow-up, depending on the availability of sufficient funds in their MediSave accounts for some of these individuals (Extract 16).

Extract 16

*As long as not using hard cash it’s okay, I don’t think she minds [going for a scan] if [the hospital is] able to deduct the medical expenses from the C[entral]P[rovident]F[und] MediSave … so if we can postpone her scan till the next year so that she can pay via her MediSave as currently for this year her MediSave is almost all used up.*

[Caregiver 3 of participant with MCI]

The financial considerations indirectly reflected how socioeconomic constraints shaped participants’ and caregivers’ decisions and behaviors about medical follow-up. Finally, commonly known problems such as long waiting times for a specialist appointment, extended clinic waiting periods, and difficulty in rescheduling a specialist appointment were acutely felt by participants and caregivers (Extracts 17 and 18). Improving these processes would make follow-up appointments for cognitive impairment less onerous and more manageable for participants and caregivers.

Extract 17

*… The problem is I couldn’t get for her [an appointment] … It is something [of a] nuisance right we have to call them and check every day … It’s not so easy, that day I call[ed] so many times they pick[ed] up and then they say (sic) no, you have to call every day. Really, it’s a very disturbing nuisance what. How can I call the hospital every day? You all put [her] on the waiting list and then it’s better for you all that … you check your computer system and then you tell us [when there is an available slot].*

[Caregiver 12 of participant with MCI]

Extract 18

*… the appointment is as good as don’t have appointment … Because we every time have to wait [for] very long … your appointment is at 2 o’clock but you’re seeing the doctor at 4 plus 5. I think it’s ridiculous cause sometimes I have to work, so I will [have] no choice try to bring my laptop with me and work in the hospital … All hospitals [are] the same, no matter where you go, you have to wait. Not just [the] hospital[s], [the] polyclinic[s] also have to wait [for a] very long [time].*

[Caregiver 18 of participant with MCI]

#### Case-Finding Result Letter as a Cue for Action

In this study, all PENSIEVE-AI participants received a letter titled “Feedback on Research Participation,” which conveyed their positive case-finding result (ie, MCI or dementia), informed them of upcoming follow-up appointments or provided instructions to arrange for a specialist appointment through a primary care doctor, and included lifestyle advice to mitigate further memory deterioration. This letter was intended to function as a physical cue, prompting participants and caregivers to take necessary follow-up actions based on the positive case-finding results. However, one caregiver reported not receiving the letter. Although this was an isolated case, it demonstrated the importance such letters can serve in prompting action or, conversely, how the absence of the result letter might contribute to inaction following a positive case-finding result. In another instance, a participant and caregiver mistakenly perceived the result letter solely as a referral letter intended for the polyclinic doctors, viewing their role as mere couriers responsible for delivering the letter to the polyclinic doctor without engaging with its content (Extract 19).

Extract 19

*… they said must go to the poly[clinic], give this letter to the poly[clinic doctor] … I didn’t actually read [this letter] properly … because I thought this is for [the] doctor, [so] I don’t want to read, I thought it might be confidential, for [the] doctor only. Now you [are] talking [to me] then I opened up and read … [previously] I asked John to bring to the doctor la (sic). So the doctor already opened up the envelope so now I’m reading [it] to you …*

[Caregiver 7 of participant with MCI] (The name (John) has been replaced with a pseudonym.)

This example illustrates that the letter’s effectiveness as a cue to prompt medical help–seeking behavior depends on participants’ and caregivers’ engagement with its content. When unread, the result letter could lose its cue effect.

#### Inadequate Physical Infrastructure to Navigate Health Care Organizations

Caregivers reported that participants often avoided using escalators due to mobility concerns (Extract 20), necessitating planning routes to medical appointments that use elevators instead.

Extract 20

*… initially, she was still okay with the escalator, stepping on the escalator that is going up. She was just afraid to step on the escalator that’s coming down. But these days, she dares not step on anything that moves.*

[Caregiver 21 of participant with dementia]

Elevators became essential in the commute, yet issues such as limited availability of elevators, lengthy wait times, and overcrowded elevators further hindered accessibility for participants and caregivers. For example, accessing elevators during peak travel hours when taking the train to medical appointments. These barriers, common to many older adults with mobility limitations, extend beyond cognitive impairment. Additional barriers include the lack of easily accessible ramps for wheelchairs and pushchairs, insufficient space for mobility devices on public transport, and poorly lit parking lots with unclear and difficult-to-find signage within health care facilities (Extract 21).

Extract 21

*the roads … are not built … for easy access or handicapped people, don't talk about even dementia people, just handicapped (sic). You see a lot of these places they are steps instead of ramps and even the ramps are not built in such a way [that is] properly designed … For the purpose of wheelchair-bound people or people having difficulty in walking, even my wife have (sic) difficulty in getting down, up and down the escalator, she gets very scared … when you are a bit older, your reaction is different. Your mental reaction … and this moving escalator is very very difficult for older people … and to be very frank as a driver I find Singapore the road … signage is very bad … The [carpark] exit, their signage is so bad … the directional signs are so bad … the underground carpark, you don't know east, west, north, south … want to go to [the] medical centre, [there is] no sign.*

[Caregiver 14 of participant with dementia]

#### Social Capital, Both an Enabler and a Barrier

Social capital, defined as the resources derived from a person’s social relationships to enable a desired social action [26], functioned as both an enabler and a barrier to medical follow-up after a cognitive impairment case-finding result. As an enabler, social capital provided direct instrumental support, such as booking or rescheduling follow-up appointments on participants’ behalf (Extract 22), reminding participants of medical appointments (Extract 23), driving and accompanying them to their appointments, and providing financial aid for medical bills (Extract 24).

Extract 22

*… my son is arranging [for his mum] to see the doctor …*

[Caregiver 14 of participant with dementia]

Extract 23

*Caregiver: I grumbled and nagged [at him to see the primary care doctor for his MCI referral letter to the specialist].*

*Participant: I cannot stand it, the nagging. That’s why [I went eventually].*

[Caregiver 7 and participant 7 with MCI]

Extract 24

*(When asked about difficulty paying for further investigation of the case-finding of cognitive impairment result) my friend always says anytime if you need financial assistance let me know.*

[Participant 5 with MCI]

Social capital also played an indirect role in facilitating timely medical follow-up among socially active participants. Social activity increased the likelihood that others with accurate knowledge about cognitive impairment would notice signs of cognitive decline and encourage medical follow-up (Extract 25).

Extract 25

*… her friend called me … during the [Japan] trip [and informed] … that she didn’t bath[e], and then personal hygiene is (sic) an issue, and then erm forgetfulness or something but then she said it’s not normal … I think your mum got to get her [to go for a] check-up because she’s behaving differently from before.*

[Caregiver 16 of participant with dementia]

This external feedback can reinforce the positive case-finding results; together, they may be more persuasive in motivating participants and their caregivers to acknowledge cognitive decline and pursue medical follow-up. Additionally, active social engagement enables participants to vicariously experience dementia in others via witnessing the lived experiences of dementia by someone they know. This exposure may heighten their sense of personal susceptibility to the disease and the importance of early medical intervention to delay the disease progression (Extracts 26 and 27).

Extract 26

*… my own sister in her 90s had advanced dementia. But hers was I think from … the age of 80 … my younger brother who was discovered after me, suddenly have (sic) the advanced dementia … and completely now couldn’t talk … he can’t recognize anybody, advanced dementia, yeah he can't control his bowel everything. Yeah so his was very fast within five years of discovering … So I don't want to follow the same route as them. In the sense that I'm aware, I can still take control of my life.*

[Participant 1 with MCI]

Extract 27

*I went to [stroke] rehab [and got to know a] man … [with] dementia … then I came to know [he was] a newspaper editor… wah very sad and pitiful when … [at] this age you have this problem … he’s educated … his mind suddenly slow[ed] down … the impact [on] normal person I don’t mind. But he’s an editor you know, that means basically he’s educated … so [you] never know, it may happen to me … the possibility [of me having dementia] is always there …*

[Participant 5 of MCI]

However, social capital can be a double-edged sword. Despite its facilitative role, it may also act as a barrier. When individuals with inaccurate knowledge about cognitive impairment normalize participants’ symptoms of cognitive decline as typical signs of aging (Extract 28), and when members of the participants’ immediate social circle (eg, primary caregivers, noncaregiving family members, friends, neighbors) disagree on the severity of the case-finding results and the importance of seeking medical attention, such discord can lead to conflict and hinder timely medical follow-up (Extract 29).

Extract 28

*I can’t remember things and my close friend … said oh no la (sic) old age la (sic) people will get old you know, will forget, yeah, so nothing la (sic) …*

[Participant 1 with MCI]

Extract 29

*… a lot of [my] neighbours, they keep telling me your father has no memory loss, dementia. See, he still can recognize us. He can still remember us, but I guess it’s because they didn’t (sic) live with him. They didn’t talk to him … if you look at him you will not think that he has dementia … So it’s actually very different [for] a caretaker and a non-caretaker, their views will be very different … it’s only when you live with them then you know that how difficult it is … [my brother who does not live with us,] it’s just like the same as the outsiders. They will say that my dad is so well but it’s only the outside, but inside?*

[Caregiver 17 of participant with dementia]

#### Caregivers With Profamily Workplace Policies

Many participants required family members to accompany them to follow-up appointments, both for instrumental support with transportation to and from the hospital and to help communicate symptoms accurately to the doctors. As many of the caregivers were still working, having a profamily employer was found to be an important facilitator that enabled them to fulfill this caregiving commitment and responsibility (Extract 30).

Extract 30

*Fortunately, my current company is quite understanding, so I just take half-day leave …*

[Caregiver 17 of participant with dementia]

#### Oversimplified Cultural Representation of Dementia

Dementia was often portrayed in mass media as involving severe symptoms such as an inability to return home or failure to recognize family members. While such portrayals increased public awareness about the disease, they inadvertently promoted the false notion that dementia was absent unless these severe symptoms were present. This misunderstanding posed as a barrier for the participants and caregivers in recognizing the seriousness of the symptoms of MCI and early-stage dementia, as daily functioning was only minimally disrupted. As a result, participants’ cognitive decline was not readily associated with dementia risk due to the mismatch with media representations. Rather than prompting timely medical help–seeking, the prevailing oversimplified cultural representation of dementia may have unintentionally justified the delays in medical follow-up (Extract 31).

Extract 31

*… she say no la no la, I only old only la (sic) … everybody [who] get[s] old [will] get this kind of forgetfulness. See, I will never get this, not like this (referring to media portrayal of advanced dementia). So she denies.*

[Caregiver 16 of participant with dementia]

### Motivation

#### Strong Affective States Triggered by the Case-Finding Results

Participants and caregivers expressed feelings of fear, fright, hopelessness, and sadness upon learning about the case-finding results. For some, these affective states acted as catalysts for seeking medical follow-up, prompting proactive actions when sufficiently intense (Extracts 32 and 33).

Extract 32

*… since COVID (sic) when I see my [cognitive] decline and my forgetfulness, that really frightens me … you (sic) seem so hopeless like there’s no cure no nothing … I see my own decline during this COVID time and then seeing my sister’s … sharp … [cognitive] decline … So that makes me worried uh scared you know … then sometimes the trouble is there is no one to point it out to you.*

[Participant 1 with MCI]

Extract 33

*Sad because I'm afraid that she can’t remember me (caregiver was tearing) … I hope she won’t reach that stage …*

[Caregiver 8 of participant with dementia]

However, for others, these affects were more ephemeral and did not spur action toward seeking medical follow-up (Extract 34).

Extract 34

*she was a bit moody for about one or two days then after that she’s back to her old self in denial again … I think [the moodiness was due to] when she do (sic) the [case-finding of cognitive impairment] test then she realized a lot of things that she cannot remember, a lot of things she cannot recall …*

[Caregiver 16 of participant with dementia]

Participants and caregivers used both emotions and moods to describe their feelings, often interchangeably, despite these being distinct constructs. Emotions, such as fear, are more concentrated, have specific sources, contain cognitive meaning, and are often short-lived, whereas moods, such as feeling bad, are relatively enduring, more diffuse, lower in intensity, lack specific sources, and have little cognitive meaning [27,28]. To capture participants’ and caregivers’ descriptions, we used the term affect as the generic psychological term that encompasses both emotions and moods [28].

#### Perceived Inaccuracy of the Case-Finding Tool

One rationale for not seeking medical attention following the positive case-finding results was rooted in the perception that the case-finding tool was biased, thus rendering the test result or diagnosis invalid. Although diagnoses in the pilot case-finding program were based on gold-standard practices (ie, consensus diagnosis by 3 specialists, including semistructured interviews, neuropsychological testing, and behavioral observations), the pilot program was embedded within a larger AI study with concurrent administration of a separate AI tool. This led to confusion among some participants and caregivers, who mistakenly assumed that the diagnosis was generated by an unvalidated AI tool still under development and testing, rather than the gold standard method for diagnosing cognitive impairment. As a result, trust in the legitimacy of the diagnosis was undermined and the need for medical follow-up was dismissed. Notwithstanding this confusion, it provided valuable insights into how perceptions of the case-finding process influence medical help–seeking behavior. Skepticism was particularly pronounced among participants with hand movement impairment due to stroke (Extract 35) and those with low technological literacy (Extract 36).

Extract 35

*I can tell you I can easily fail [the case-finding of cognitive impairment test] … [as my mobility is] left [with] only the left hand … I think … I’m not able to pass … Because of my handicap [due to stroke] …*

[Participant 5 with MCI]

Extract 36

*She wasn’t able to answer the questions because of the [case-finding tool] technology, she doesn’t know how to use [the tool] … we don’t know the [diagnostic] accuracy of the [case-finding] tool. But the brain scan is very accurate … which we haven’t done.*

[Caregiver 3 of participant with MCI]

In an extreme case, the case-finding process per se was perceived to be the direct cause of the MCI or dementia result (Extract 37).

Extract 37

*I guess that she doesn't want to know her condition … she ever commented … the more you test, the more you see all this sickness, the better not to know that kind … Don't test, don't know, don't have.*

[Caregiver 16 of participant with dementia]

#### Valuing Proactive Medical Disease Management

Several reasons were identified that facilitated timely medical follow-up after a positive case-finding result. These included the adage that prevention is better than cure, obtaining professional medical advice on disease management, and close monitoring of disease progression by a medical professional (Extract 38).

Extract 38

*I don’t see it as unnecessary [to see the doctor even though there is no treatment] … the specialists … at least [can] check … whether her condition deteriorates or improves or whatever … I don’t see it as pointless because we’re not doctors, we’re not specialized, you (sic) don’t even know how to evaluate the situation and her situation. So I think it would be good if the doctor in their professionalism they can actually advise her what is the condition, or at least we are prepared [for] what is going to happen. Rather than you just judge by yourself, you don’t know whether it is right or wrong [and] make the wrong judgment.*

[Caregiver 18 of participant with MCI]

Participants and caregivers also sought professional medical advice to support practical future planning and informed decision-making ahead of further disease deterioration. This included making a Lasting Power of Attorney to organize affairs and downsizing to a smaller apartment with dementia-friendly facilities to better accommodate the needs of the family member with dementia (Extract 39).

Extract 39

*It is still [a] doubt or maybe [to hear from the doctor] mention by at what stage or what? … Simple things like … how? Should we downsize [our house or] relocate? And then [if we do] relocate, what kind of facilities should we look for to deal with the dementia patient? We have [a] general idea, but then we need something even more concrete [like concrete advice from the doctors] … and then which route to take or the best route to take, [what are the] possible route[s] to take. And then from there we will say, okay the route we could change later on but then at least how do we get started regarding the new route …*

[Caregiver 14 of participant with dementia]

#### Lay Beliefs About Seeking Medical Care When Sick

The term “lay beliefs” is used here to refer to participants’ and caregivers’ understandings of when medical attention is needed. These beliefs may differ from clinical recommendations that promote timely and appropriate medical help–seeking for cognitive concerns. Rather than being uninformed or irrational, such beliefs are shaped by the personal, social, cultural, and economic contexts. Within this interpretive frame, several lay beliefs about medical help–seeking when sick were identified. Among participants who defaulted on their medical follow-up, there was a common belief that one should only visit the doctor when severely sick. Here, severe sickness is defined as a medical condition that markedly interferes with daily living and functional abilities (Extract 40).

Extract 40

*For her current condition, I don’t think there is a need for her to go for a brain scan … Because the doctor told me there is mild, moderate, and severe for this condition. To me, her mild condition isn’t affecting her life much. But if I were to notice her condition worsens, then yes, even if have to pay cash for the [brain] scan we would go … How to differentiate mild, moderate, and severe … if for example … she went out via M[ass] R[apid] T[ransit] (train network) and can return home on her own, I think it’s okay. But if she is unable to return home on her own, then I think it is problematic … and we must take action [to see the doctor].*

[Caregiver 3 of participant with MCI]

Because MCI and early dementia often progress insidiously and may not noticeably disrupt daily living and functional abilities in the initial stages, some participants and caregivers perceived seeking medical attention for such conditions as unnecessary or even financially wasteful, particularly in the context of Singapore’s high cost of living and the considerable out-of-pocket expenses associated with consulting a primary care doctor (Extract 41).

Extract 41

*… I just wanted to bring her to the polyclinic for referral to [the] hospital but she is in denial … She says she doesn't [want treatment] … she’s quite independent, quite stubborn, so I cannot force her to do anything … she said it’s a waste of money [to see doctor] … in general. So she’s kind of averse to seeing a doctor because nowadays seeing the G[eneral] P[ractitioner] is not cheap …*

[Caregiver 16 of participant with dementia]

This belief was particularly salient among participants managing comorbid conditions (eg, cancer, cardiovascular diseases, and respiratory diseases), which were perceived as more severe, urgent, or life-threatening than cognitive impairment (Extracts 42 and 43).

Extract 42

*… there is nothing worse than cancer … When people get sick, very sick or what, it’s okay … What I mean is that when people die, people got cancer or what, take it easy lah. No need to worry [about the problems associated with MCI] …*

[Participant 4 with MCI]

Extract 43

*It’s more of his asthma and his episodes of the asthma … it’s more of his concern [than MCI] …*

[Caregiver 6 of patient with MCI]

Among participants with comorbidities, disease prioritization was shaped by subjective interpretations of illness severity and financial considerations. Although these lay beliefs may diverge from clinical guidelines and recommendations advocating for early detection and intervention for cognitive impairment, they reflect a coherent lay understanding of disease severity, disease burden, and the health priorities, shaped by the lived experiences and concerns of both participants and caregivers.

## Discussion

### Principal Findings

This qualitative study identified 15 barriers and facilitators to medical help–seeking behavior following a diagnosis of MCI or dementia, categorized using the COM-B model. The discussion first contextualizes the findings within the existing literature, followed by integrated evidence- and practice-based recommendations.

### Barriers

Our analyses on inaccurate knowledge about MCI and dementia, inability to recognize the symptoms of MCI and dementia, complicated health care processes, and low health literacy to navigate the health care processes aligned with commonly reported themes in the literature, including normalization of problems, lack of knowledge about dementia, and the complexity and inaccessibility of health care services [12,16,29,30]. Lay beliefs about seeking medical care when sick mirrored the finding of the review by Hill et al [30] on the perceived lack of benefit in medical help–seeking. Both reflect mismatches between clinical recommendations and the lay understandings that can undermine the perceived value of medical treatment, highlighting the need to increase public awareness of the benefits of timely medical intervention and care.

Strong affective states triggered by positive case-finding results were consistent with the findings of Jiao et al [16], who noted that individuals diagnosed with MCI perceived the disease as inherently threatening, characterized by negative valence emotions, such as anxiety and fear regarding disease progression. While Devoy and Simpson [29] identified fear as a barrier to medical help–seeking, our findings suggest a more nuanced, dual role, in which fear, conceptualized as a strong affective state triggered by the case-finding results, functions as both a facilitator and a barrier (eg, Extract 32). This duality reflects the multidimensional nature of fear, encompassing affective, cognitive, and psychobiological dimensions [31], with each influencing medical help–seeking behavior in different ways [32]. In the context of cancer screening, for instance, the cognitive and affective dimensions of fear facilitated cancer screening participation, while the psychobiological dimension hindered it [32].

A unique barrier identified in this study was the perceived inaccuracy of the case-finding tool. As participants were recruited from the larger case-finding pilot program using an AI tool, concerns about the tool’s accuracy arose despite participants receiving diagnoses through the gold-standard consensus method. Future implementation of the PENSIEVE-AI tool [11] will need to explicitly address accuracy concerns and clarify the role of the AI tool in the cognitive impairment diagnosis process, particularly given that the consensus approach by memory specialists is not typical in routine clinical practice.

Medical help–seeking was further complicated by anosognosia in individuals with dementia and symptom fluctuation among individuals with MCI. Anosognosia, the lack of insight into one’s own cognitive impairment, is a characteristic of dementia [33] that may contribute to denial and resistance to medical follow-up. This is evident in Extracts 10 and 41, where a caregiver described how her mother was in denial and misattributed cognitive symptoms to normal aging, features potentially indicative of the presenting symptoms of anosognosia. Similarly, among individuals with MCI, a clinically and neuropathologically heterogeneous group [34], the progression to dementia varies; while approximately 46% of individuals with MCI aged 65 years and older progress to dementia within 3 years [35], rising to 65% for those aged 75 years and older [36], others reported symptom improvement [37,38]. This symptom fluctuation makes early signs of cognitive impairment harder to recognize, contributing to uncertainty and delays in seeking medical help, as reflected in Extracts 7, 11, and 29.

### Facilitators

Despite the identified barriers, several facilitators supported medical help–seeking behaviors. These included the ability to recognize cognitive impairment symptoms and the adoption of practical strategies to navigate symptom-related challenges, such as tracking medical appointments. Strong social capital, particularly reliance on caregivers and friends to reschedule medical appointments, provide transportation, and accompany individuals to medical appointments, facilitated medical follow-up behavior. Additionally, caregivers with profamily workplace policies and the case-finding result letter served as a physical cue to prompt medical follow-up action. For some individuals diagnosed with cognitive impairment, the strong affective states triggered by the case-finding results also prompted medical follow-up, particularly among those who valued proactive disease management through medical professional monitoring of disease progression. These facilitators align with the literature highlighting the importance of positive social support, disease knowledge, and the value of early detection in promoting medical help–seeking [16,29,30].

Our findings contrast with the paradoxical finding of Jiao et al [16] that individuals with MCI were less likely to seek medical help when using cognitive strategies. Our study found that individuals with MCI actively adopted strategies to track medical appointments, reflecting problem-solving efforts by the diagnosed individuals and their families to manage cognitive impairment symptoms. Notably, those with strong social capital and support networks were able to overcome some of the current barriers identified in this study, including physical impairments, low literacy to navigate health care processes, inadequate physical infrastructure, complicated health care processes, and lay beliefs about seeking medical care when sick. Facilitators unique to this study included caregivers with profamily workplace policies and the case-finding results letter as a cue for action. The former strengthened social capital, while the latter is a distinctive finding in this qualitative study on determinants of medical help–seeking behavior following a cognitive impairment diagnosis via a community-based AI case-finding tool.

### Integration With the Alzheimer Disease System Framework

Our findings extend the Alzheimer disease (AD) system framework of Ball et al [39] by specifying the barriers and facilitators influencing medical help–seeking behavior following a cognitive impairment diagnosis. These determinants can be situated within the AD system framework, between “health care system patient flow” and “access to support, care, and delivery,” specifically following the “timely and accurate diagnosis” node [39]. Notably, current results demonstrate that the pathway from cognitive impairment case-finding or diagnosis to medical help–seeking is nonlinear. Instead, it is a process punctuated by interrelated determinants at multiple junctures, through a series of interactions between individual capacities and motivations, physical and social dynamics and opportunities, and broader structural conditions.

Although COM-B provides a systematic structure for identifying these determinants, several exhibit cross-domain interdependence. For instance, while physical impairments hindering medical follow-up are categorized under the physical capability subdomain, this barrier is closely linked to the social opportunity subdomain; many participants often relied on caregivers for transportation and company, a form of social capital that is both an enabler and a barrier. Such overlaps highlight the complexity and dynamic nature of medical help–seeking behavior following cognitive impairment case-finding results. Recognizing these interplays is essential for a nuanced understanding of medical help–seeking behavior and for designing interventions that address barriers within their broader systemic and contextual realities rather than in isolation.

### Actionable Recommendations

In view of this complexity, we developed actionable recommendations by mapping BCW intervention functions onto our analytic findings. In addition to barriers, facilitators across the COM-B domains were integrated to bolster support for timely medical follow-up among individuals with cognitive impairment. Each determinant was mapped to one or more BCW intervention functions, as detailed in the matrix in Table 2. Training, coercion, and restrictions of the behavior change intervention functions were excluded; training addresses knowledge and skill gaps that are already covered by the education and enablement intervention functions, while coercion and restrictions are not suitable in this context. Further details of the recommendations are provided in Textbox 1.

**Table 2.** Matrix of the key determinants mapped onto the capability, opportunity, motivation-behavior (COM-B) domains and the suggested behavior change intervention functions.

Key determinants mapped onto COM-B^a^ domains	Behavior change intervention functions^b^					
	Education	Persuasion	Role modeling	Enablement	Incentivization	Environmental restructuring
Capabilities						
Physical impairments can hinder medical follow-up			✓	✓	✓	✓
Adopting strategies to track medical appointments	✓		✓	✓		
Low health literacy to navigate the health care processes	✓		✓	✓		
Inaccurate knowledge about MCI^c^ and dementia	✓	✓		✓		
(In)ability to recognize symptoms of MCI and dementia	✓	✓		✓		
Opportunities						
Complicated health care processes				✓	✓	
Case-finding result letter as a cue for action		✓		✓		
Inadequate physical infrastructure to navigate health care organizations				✓		✓
Social capital, an enabler and a barrier	✓	✓	✓	✓	✓	
Caregivers with profamily workplace policies					✓	
Oversimplified cultural representation of dementia	✓		✓			
Motivation						
Strong affective states triggered by the case-finding results	✓	✓	✓	✓		
Perceived inaccuracy of the case-finding tool		✓		✓		
Valuing proactive medical disease management	✓	✓	✓		✓	
Lay beliefs about seeking medical care when sick	✓	✓	✓	✓		

a COM-B: capability, opportunity, motivation-behavior.

b Training, coercion, and restrictions of the behavior change intervention functions are excluded.

c MCI: mild cognitive impairment.

Textbox 1. **Actionable recommendations based on the behavior change intervention functions.**

**Education** through promoting awareness and understanding of early cognitive impairment among the public, consumers, and health care professionals is pivotal. Educational efforts can be delivered through public outreach initiatives (eg, television programs, hospital websites, public roadshows, newsletters, informational brochures included with the case-finding result letters) with a focus on the following:

1. **Recognizing symptoms**: educating individuals about the symptoms of mild cognitive impairment (MCI) and the various stages of dementia to enable early recognition of cues and to seek timely medical help.
2. **Future planning**: highlighting the importance and value of early planning for care and decision-making for the present and the future to motivate individuals to address cognitive health.
3. **Reframing MCI and dementia as a cognitive disability**: reframing cognitive impairment, as cognitive disability as this is not different from physical disabilities. Such reframing can plausibly destigmatize cognitive impairment and rally a whole-of-society approach to improve medical help–seeking behavior. Individuals may be more likely to seek help when cognitive impairment is not perceived as a liability (ie, akin to a physical disability that requires prostheses to maintain normalcy), while immediate family and the rest of society will be more willing to step up to provide support to individuals with cognitive impairment, akin to providing a prosthetic environment for the cognitive disability.
4. **Facilitating conversations**: strategies for broaching the topic of medical help–seeking with loved ones who are resistant to medical help–seeking for their cognitive impairment (eg, expressing concern, validating feelings, addressing misconceptions, and collaboratively exploring alternatives).

**Persuasion** through the following strategies:

1. **Providing result letters after case-finding efforts**: the letters should be designed using psychological and behavioral science theories and principles to encourage medical follow-up. The letters should:

- Clearly explain the benefits of further follow-ups and diagnostic tests, such as brain imaging, to rule out cognitive impairments that may be treatable.
- Highlight the various subtypes of cognitive impairment, including those that may be treatable or reversible.
- Include personalized risk of dementia to increase the urgency of the desired action.
- Provide a clear, concise, and easy-to-understand description of the follow-up procedures, including the estimated cost after government subsidies, and the expected duration of each procedure.
- Highlight the benefits of early diagnosis and management of cognitive impairment, which extend beyond direct immediate health benefits, such as enabling individuals with cognitive impairment to secure a better quality of life with the necessary help and support and allowing them to plan for the future and prioritize what matters most to them even in the face of cognitive impairment.

1. **Featuring dementia specialists and community leaders on television programs and radio talk shows.**

**Role modeling** by showcasing interviews and testimonials from individuals who sought medical help for early cognitive impairment and are living physically, mentally, and socially active lifestyles can serve as powerful role models. These interviews should ideally incorporate the following key topics:

1. **Rationale for proactive disease management**: their reasons for taking a proactive approach to managing their condition.
2. **Support systems**: the support they received from health care professionals, caregivers, friends, and the community.
3. **Strategies to cope and live with the disease**: how they manage the strong negative emotions triggered by the case-finding results and the strategies they use to keep track of their medical appointments.

Interactive and social media platforms can be used to disseminate these videos, fostering a more proactive approach to medical care.

**Enablement** through:

1. **Providing information on frequently asked questions about the case-finding tool**: addressing confusion, doubts, and skepticism of the validity of the case-finding results by offering clear and accessible information on the frequently asked questions.
2. **Simplifying the referral process via direct referrals to memory clinics**: streamlining the referral process to increase follow-up rates and minimize delays by implementing direct referrals to specialized memory clinics.
3. **Formulating practice guidelines**: the practice guidelines should include simplified key points on when primary care physicians should be concerned about cognitive impairment (as opposed to normal aging) and simplified criteria on when referrals to memory clinics are considered appropriate.
4. **Deploying trained community workers/volunteers** (eg, from Active Ageing Centres and Silver Generation Office) for engagement with individuals with positive case-finding results. These community workers/volunteers can serve the dual purpose of monitoring well-being and providing educational and persuasive interventions and support, including:

- Reinforcing the importance of seeking medical help for early cognitive impairment to both individuals and caregivers.
- Supporting individuals in managing the strong negative affective states triggered by the case-finding results.
- Guiding individuals and caregivers on the strategies for tracking medical appointments.
- Coaching caregivers on managing resistant behaviors.
- Assisting with rescheduling of medical follow-up appointments if necessary.
- Accompanying individuals to the memory clinics, particularly those without immediate caregivers.
- Escalating more complex behaviors or challenges to the community health care workers (eg, community nurses).
- Linking individuals to available community programs that address the risk factors for cognitive decline (eg, programs that target physical activities, diet, and cognitive and social engagement).

**Incentivization** is particularly important given that financial concerns and socioeconomic constraints can deter medical help–seeking behavior. Strategies should therefore prioritize affordability and equity, including policies aimed at reducing out-of-pocket costs and addressing indirect financial burdens to improve access for individuals and families with constrained financial resources. Incentivization can be implemented in the form of:

1. **Financial incentives for medical follow-up**: providing financial incentives to individuals with positive case-finding results to encourage attending memory clinics (eg, indirect financial rewards).
2. **Increasing the withdrawal limit for national medical savings**: increasing the annual withdrawal limit of health insurance or MediSave for diagnostic testing thereby reducing out-of-pocket payment for diagnostic imaging and subsequent treatments.
3. **Subsidy for initial consultation fees**: waiving the consultation fee for the initial visit to memory clinics or providing a higher subsidy for specialist follow-ups.
4. **Providing subsidized transportation**: offering subsidized transportation to memory clinics, particularly for individuals who have physical difficulties. This should also include the accompanying caregiver and community volunteer.
5. **Profamily workplace policies** as a type of nonfinancial incentive by providing work-from-home options for caregivers, additional leave for caregiving responsibilities, increased sick leave for individuals with MCI and dementia, and emergency time off (eg, when an individual with dementia gets lost and fails to return home).

**Environmental restructuring** to improve accessibility, safety, and user-friendliness:

1. **Wheelchair and pushchair accessibility**: improving wheelchair and pushchair accessibility (eg, using elevators instead of escalators, and ramps instead of steps) in hospitals, public spaces, and public transport.
2. **Clear and prominent signage**: installing clear and prominent signage in public spaces, public transport, and memory clinics to aid wayfinding.
3. **Wheelchair-friendly toilets**: increasing the availability of wheelchair-friendly toilets in public spaces, near memory clinics, and at train stations.

The education behavior change intervention function addresses barriers related to psychological capability, social opportunity, and both automatic and reflective motivation. Consistent with Ball et al [39], educational content should be tailored to different audiences (ie, the general public, consumers, and health care professionals). Points 1-3 outlined in Textbox 1 apply across all audiences, whereas Point 4 targets the general public and health care professionals. Building on evidence showing improved public dementia knowledge and attitude [40], education efforts, public campaigns, and media should evolve beyond depicting individuals with severe dementia to include individuals living with MCI and early stages of dementia. In doing so, they can illustrate how individuals adapt and continue to live positively and meaningfully despite cognitive decline. This shift will enable the public to gain a more accurate understanding of early cognitive impairment, emphasizing that dementia is more than just memory loss; it also involves language difficulties, problem-solving challenges, and changes in mood and behavior. By providing a broader perspective, it can increase psychological capability and reflective motivation, thereby encouraging timely medical help–seeking behavior even in the early stages of the disease.

In tandem with education, persuasion strategies can activate both automatic and reflective motivation processes. For individuals reluctant to seek medical help after receiving positive case-finding results, carefully designed result letters may serve as a behavioral nudge, drawing on psychological and behavioral science principles, such as nudges [41-43] and loss-framed messaging that emphasizes the negative consequences of delaying medical follow-up [44]. Point 2, engaging the public through expert figures and community leaders to offer insights and perspectives on early cognitive impairment, is a powerful persuasion strategy that draws on the principle of authority to engage the public and encourage medical help–seeking behavior by deferring to the experts (eg, memory specialists) [45,46], and positioning these experts as catalysts for behavior change.

The enablement intervention function is necessary to reduce structural and procedural barriers. Our study supports simplifying the referral pathways; participants receiving direct referrals to SGH memory clinics achieved a 100% attendance rate (ie, for those living in the SGH vicinity, n=10), compared to 54.6% for those with indirect referrals to memory clinics within their home vicinity (ie, participants living outside of the SGH vicinity, n=6). The differences point to the value of simplifying the referral process to reduce cognitive load and barriers in navigating the health care system. At the organizational and policy level, establishing clear practice guidelines for referrals from primary care to memory clinics would reduce practice variations across the health care system and improve the opportunity to timely access medical care.

Finally, environmental restructuring is essential to address physical opportunity barriers, particularly for individuals living with mobility challenges. Limitations in Singapore’s public transport can hinder attendance at follow-up medical appointments, particularly during peak commuting hours when public transport is crowded and less accessible. For instance, the Singapore public buses are only able to accommodate one or 2 wheelchair users at a time [47], requiring additional wheelchair users to wait for the next available bus. Environmental restructuring and structural modifications, such as increasing wheelchair capacity on buses and improving clinic accessibility, would not only ease travel burdens but also signal a societal commitment to inclusion by proactively supporting the diverse needs of people living with cognitive and physical impairments.

### Limitations and Strengths

This study has several limitations. First, the sample was drawn from participants in the PENSIEVE-AI study [11], raising the possibility of self-selection bias whereby participants may have been more inclined to follow up on their cognitive impairment. To mitigate this, we purposively included individuals who had not sought medical follow-up and expressed no intention to do so unless their symptoms became more severe (eg, difficulty returning home using a familiar route). Second, the sample lacked ethnic diversity, with limited representation from Malay and Indian minority groups relative to the Chinese majority group. Third, the long intervals between memory clinic follow-ups, typically 6-12 months, introduce uncertainty regarding continued long-term medical follow-up, particularly for individuals with MCI whose symptoms are typically less severe and may be perceived as less disruptive than those associated with dementia.

A further limitation concerns the exclusion of individuals with dementia from direct interviews. Although based on ethical and practical considerations regarding potential distress and communication challenges, this warrants critical reflection on the representation gap inherent in relying on proxy accounts. This reliance placed emphasis on caregivers’ experiences and perspectives in identifying barriers and facilitators of the medical help–seeking journey following dementia diagnosis, inadvertently glossing over the affective experiences and personal autonomy of the individuals with dementia themselves. In particular, their personal decision-making in medical help–seeking, sense-making of the dementia diagnosis, and challenges in navigating health care services, as well as the interactions with caregivers and health care professionals may be underrepresented, despite being highlighted in the broader dementia literature [48-50].

Prior research emphasizes the importance of including individuals with dementia to address epistemic injustice and support person-centered approaches in research [51-56]. While caregiver accounts were sufficiently informative for understanding the barriers and facilitators to medical help–seeking in this study, we acknowledge the importance and value of including the perspectives of individuals with dementia. Future studies should adopt more inclusive and adapted approaches, such as process consent, to ethically and effectively engage individuals with dementia. Finally, evaluating the feasibility and effectiveness of our recommendations was beyond the scope of this qualitative study. Future research should apply the acceptability, practicability, effectiveness, affordability, side-effects, and equity (APEASE) criteria [19] in collaboration with stakeholders, including policymakers and community representatives (eg, patient advocates, representatives from caregiver organizations, community health care workers, or volunteers), to refine and evaluate these recommendations.

Despite these limitations, this study makes several important contributions. First, while prior research has primarily focused on medical help–seeking intentions [12,29], this study explored determinants of actual medical help–seeking behavior following a cognitive impairment diagnosis. Second, by including individuals with MCI and the caregivers of individuals with MCI or dementia who had not sought medical follow-up, we addressed a key sampling gap in earlier qualitative work [16]. This provided a more nuanced understanding of the determinants of medical help–seeking behavior, particularly among those not yet engaged in follow-up care. In doing so, this study extends the AD system framework [39] by highlighting the nonlinear processes involved in seeking medical treatment.

### Conclusions

This study identified key barriers and facilitators to medical help–seeking behavior following a cognitive impairment diagnosis, demonstrating the dynamic and nonlinear processes in accessing medical treatment. Building on these findings, we developed actionable recommendations grounded in the COM-B framework and the BCW intervention functions. We advocate for a whole-of-society approach, with implications extending to policymakers, primary care providers, tertiary hospitals, and the transport and media authorities. To maximize the impact of these recommendations, ongoing collaboration with stakeholders and careful evaluation using the APEASE criteria are needed to address practical considerations and promote equity in facilitating timely medical follow-up after positive case-finding results or diagnosis.

## Supplementary material

10.2196/79386 Multimedia Appendix 1 Flowchart of participants in PENSIEVE-AI tool case-finding pilot study.

10.2196/79386 Multimedia Appendix 2 Interview guide using the capability, opportunity, motivation-behavior (COM-B) framework.

## Abbreviations

AD: Alzheimer disease
AI: artificial intelligence
APEASE: acceptability, practicability, effectiveness, affordability, side-effects, equity
BCW: behavioral change wheel
COM-B: capability, opportunity, motivation-behavior
COREQ: Consolidated Criteria for Reporting Qualitative Research
MCI: mild cognitive impairment
SGH: Singapore General Hospital

## References

[1] GBD 2019 Dementia Forecasting Collaborators (2022). Estimation of the global prevalence of dementia in 2019 and forecasted prevalence in 2050: an analysis for the Global Burden of Disease Study 2019. The Lancet.

[2] Prince M, Bryce R, Ferri C (2011). World alzheimer report 2011: the benefits of early diagnosis and intervention. Alzheimer’s Disease International.

[3] Morley JE, Morris JC, Berg-Weger M (2015). Brain health: the importance of recognizing cognitive impairment: an IAGG consensus conference. J Am Med Dir Assoc.

[4] Burns A, Iliffe S (2009). Dementia. BMJ.

[5] Thyrian JR, Hertel J, Wucherer D (2017). Effectiveness and safety of dementia care management in primary care: a randomized clinical trial. JAMA Psychiatry.

[6] Vickrey BG, Mittman BS, Connor KI (2006). The effect of a disease management intervention on quality and outcomes of dementia care: a randomized, controlled trial. Ann Intern Med.

[7] Cepoiu-Martin M, Tam-Tham H, Patten S, Maxwell CJ, Hogan DB (2016). Predictors of long-term care placement in persons with dementia: a systematic review and meta-analysis. Int J Geriatr Psychiatry.

[8] Jennings LA, Laffan AM, Schlissel AC (2019). Health care utilization and cost outcomes of a comprehensive dementia care program for medicare beneficiaries. JAMA Intern Med.

[9] Spijker A, Vernooij-Dassen M, Vasse E (2008). Effectiveness of nonpharmacological interventions in delaying the institutionalization of patients with dementia: a meta-analysis. J Am Geriatr Soc.

[10] Jia L, Du Y, Chu L (2020). Prevalence, risk factors, and management of dementia and mild cognitive impairment in adults aged 60 years or older in China: a cross-sectional study. Lancet Public Health.

[11] Liew TM, Foo JYH, Yang H (2025). PENSIEVE-AI a brief cognitive test to detect cognitive impairment across diverse literacy. Nat Commun.

[12] Gregg JE, Simpson J, Nilforooshan R, Perez-Algorta G (2021). What is the relationship between people with dementia and their caregiver’s illness perceptions post-diagnosis and the impact on help-seeking behaviour? A systematic review. Dementia (London).

[13] Conner M, Norman P (2022). Understanding the intention-behavior gap: the role of intention strength. Front Psychol.

[14] Faries MD (2016). Why we don’t “Just Do It”. Am J Lifestyle Med.

[15] Sheeran P, Webb TL (2016). The nitention–behavior gap. Soc Personal Psych.

[16] Jiao YC, Chang J, Liu C, Zhou SY, Ji Y, Meng Y (2023). Factors influencing the help-seeking behavior in patients with mild cognitive impairment: a qualitative study. BMC Health Serv Res.

[17] Glanz K, Rimer BK, Viswanath K (2015). Health Behavior: Theory, Research, and Practice.

[18] Michie S, van Stralen MM, West R (2011). The behaviour change wheel: a new method for characterising and designing behaviour change interventions. Implementation Sci.

[19] West R, Michie S, Atkins L (2019). Achieving behaviour change: a guide for local government and partners. Public Health England.

[20] Gong N, Yang D, Zou J (2023). Exploring barriers to dementia screening and management services by general practitioners in China: a qualitative study using the COM-B model. BMC Geriatr.

[21] Michie S, Atkins L, West R (2014). The Behaviour Change Wheel: A Guide to Designing Interventions.

[22] Tong A, Sainsbury P, Craig J (2007). Consolidated criteria for reporting qualitative research (COREQ): a 32-item checklist for interviews and focus groups. Int J Qual Health Care.

[23] Gale NK, Heath G, Cameron E, Rashid S, Redwood S (2013). Using the framework method for the analysis of qualitative data in multi-disciplinary health research. BMC Med Res Methodol.

[24] Babbage D, Terry G (2025). Thematic analysis coding management macro.

[25] What is health literacy?. Centers for Disease Control and Prevention.

[26] Kawachi I, Kennedy BP, Glass R (1999). Social capital and self-rated health: a contextual analysis. Am J Public Health.

[27] Forgas JP, Zanna MP (1992). Advances in Experimental Social Psychology.

[28] Forgas JP (1995). Mood and judgment: the affect infusion model (AIM). Psychol Bull.

[29] Devoy S, Simpson EEA (2017). Help-seeking intentions for early dementia diagnosis in a sample of Irish adults. Aging Ment Health.

[30] Hill NL, Bratlee-Whitaker E, Sillner A, Brautigam L, Mogle J (2021). Help-seeking for cognitive problems in older adults without dementia: a systematic review. Int J Nurs Stud Adv.

[31] Öhman A, Lewis M, Haviland-Jones JM, Barrett LF (2008). Handbook of Emotions.

[32] Vrinten C, Waller J, von Wagner C, Wardle J (2015). Cancer fear: facilitator and deterrent to participation in colorectal cancer screening. Cancer Epidemiol Biomarkers Prev.

[33] Newens AJ, Forster DP, Kay DW (1994). Referral patterns and diagnosis in presenile Alzheimer’s disease: implications for general practice. Br J Gen Pract.

[34] Cooper C, Sommerlad A, Lyketsos CG, Livingston G (2015). Modifiable predictors of dementia in mild cognitive impairment: a systematic review and meta-analysis. Am J Psychiatry.

[35] Tschanz JT, Welsh-Bohmer KA, Lyketsos CG (2006). Conversion to dementia from mild cognitive disorder: the Cache County Study. Neurology (ECronicon).

[36] Busse A, Angermeyer MC, Riedel-Heller SG (2006). Progression of mild cognitive impairment to dementia: a challenge to current thinking. Br J Psychiatry.

[37] Mitchell AJ, Shiri-Feshki M (2008). Temporal trends in the long term risk of progression of mild cognitive impairment: a pooled analysis. J Neurol Neurosurg Psychiatry.

[38] Mitchell AJ, Shiri-Feshki M (2009). Rate of progression of mild cognitive impairment to dementia--meta-analysis of 41 robust inception cohort studies. Acta Psychiatr Scand.

[39] Ball DE, Mattke S, Frank L (2023). A framework for addressing Alzheimer’s disease: without a frame, the work has no aim. Alzheimers Dement.

[40] Hansra GK, Lim H, Cheong CY, Yap P (2023). Knowledge and attitudes towards dementia among the general public in Singapore: a comparative analysis. Dement Geriatr Cogn Disord.

[41] (2024). Levers of behavior change: a guide to the science and applications. Rare’s Center for Behavior & the Environment.

[42] Samson A The behavioral economics guide 2024.

[43] Sunstein CR (2014). Nudging: a very short guide. J Consum Policy.

[44] Tannenbaum MB, Hepler J, Zimmerman RS (2015). Appealing to fear: a meta-analysis of fear appeal effectiveness and theories. Psychol Bull.

[45] Cialdini RB (2001). Harnessing the science of persuasion. Harv Bus Rev.

[46] Cialdini RB (2001). The Science of Persuasion. Sci Am.

[47] Special assistance. Singapore Bus Services Transit.

[48] Górska S, Forsyth K, Maciver D (2018). Living with dementia: a meta-synthesis of qualitative research on the lived experience. Gerontologist.

[49] Mammen JR, Goldman JG, Tyo M, Xiao Y (2025). Examining the lived experience of dementia with Lewy bodies through qualitative research: a systematic review. Alzheimers Dement.

[50] Prorok JC, Horgan S, Seitz DP (2013). Health care experiences of people with dementia and their caregivers: a meta-ethnographic analysis of qualitative studies. CMAJ.

[51] Beuscher L, Grando VT (2009). Challenges in conducting qualitative research with individuals with dementia. Res Gerontol Nurs.

[52] Diaz-Gil A, Brooke J, Kozlowska O, Jackson D, Appleton J, Pendlebury S (2023). A human rights-based framework for qualitative dementia research. Nurs Ethics.

[53] Halonen U, Aaltonen M, Aerschot LV, Pirhonen J (2025). Participation of persons living with dementia in research: a means to address epistemic injustice. Dementia (London).

[54] McKeown J, Clarke A, Ingleton C, Repper J (2010). Actively involving people with dementia in qualitative research. J Clin Nurs.

[55] Murphy K, Jordan F, Hunter A, Cooney A, Casey D (2015). Articulating the strategies for maximising the inclusion of people with dementia in qualitative research studies. Dementia (London).

[56] Novek S, Wilkinson H (2019). Safe and inclusive research practices for qualitative research involving people with dementia: a review of key issues and strategies. Dementia (London).

