# Supplementary figures and images for: Determinants of Medical Help–Seeking Behavior Following Case Finding of Early Cognitive Impairment: Semistructured Interview Study of Patients and Caregivers
Source: JMIR Aging. 2026 May 19;9:e79386. doi: 10.2196/79386 (PMC13186523; doi:10.2196/79386)

**Appendix 1**

**Figure 1.** Flowchart of participants in PENSIEVE-AI tool case-finding pilot study [11].


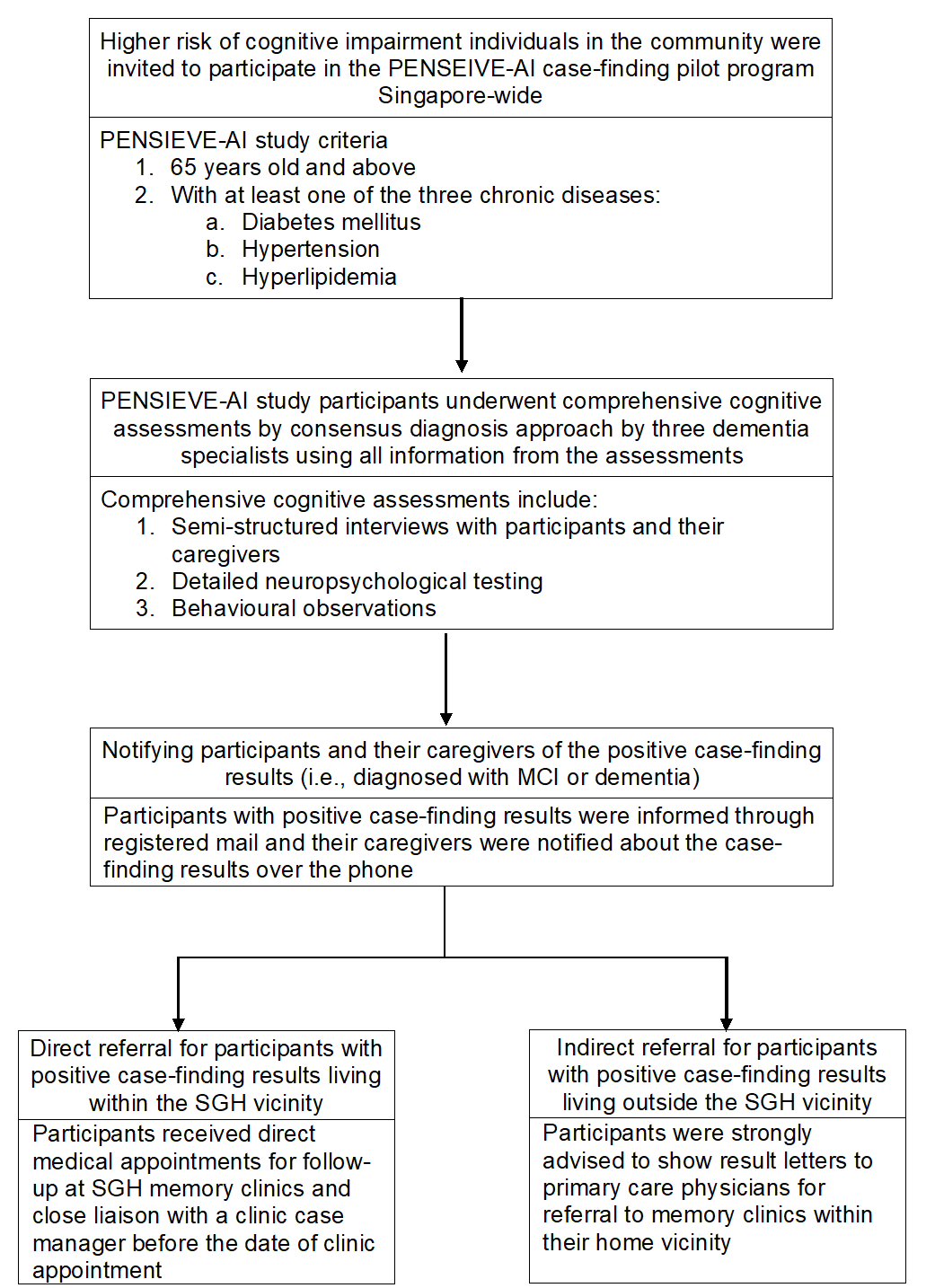

Supplement: Multimedia Appendix 1 [file aging-v9-e79386-s001.docx]
