# Supplementary material for: Determinants of Medical Help–Seeking Behavior Following Case Finding of Early Cognitive Impairment: Semistructured Interview Study of Patients and Caregivers
Source: JMIR Aging. 2026 May 19;9:e79386. doi: 10.2196/79386 (PMC13186523; doi:10.2196/79386)
Supplement: Multimedia Appendix 2 [file aging-v9-e79386-s002.docx]

**Appendix 2.**

Interview guide using the COM-B framework [18].

This version is for patient participants who were diagnosed with MCI. If only caregivers were interviewed (i.e. for patient participants who were diagnosed with dementia), the word “you” was replaced with “your loved one” throughout the interview guide.

| 1. How did you feel when you received your diagnosis of dementia/MCI through the recent PENSIEVE study? (*automatic motivation*) |
| --- |
| 1. Can you share with me what is your understanding of dementia/MCI? (*psychological capability*)    1. How did you learn about this condition? (*physical and social opportunity*)       1. Did you get them from e.g., TV programs, news, campaigns, famous people, families and friends? Could you tell me more about them?       2. How did these affect your decision to seek treatment?    2. Do you know anyone with dementia/MCI? (*social opportunity*)   **If yes,**   - - 1. What is his/her relationship with you?     2. Can you share with me what it is like to have dementia based on your observation of [name of relationship]’s life?     3. how did that affect your decision to seek treatment? (*reflective motivation*) |
| 1. What actions did you take after learning about your diagnosis through the PENSIEVE study? (*reflective motivation*)    1. Tell me more about what you did.    2. Did you discuss your diagnosis with your family and friends? (*social opportunity*)       1. What were their reactions?       2. How did you feel about their reactions? (*automatic motivation*) |
| 1. Now I’d like to ask you a few questions specifically about seeing a doctor for your dementia/MCI diagnosis. First, are you currently seeing a doctor for your dementia/MCI diagnosis? (*physical capability*)   **If seeing the doctor**   - 1. What motivated you to see the doctor? (*automatic and reflective motivation*)   2. How easy/difficult was it for you to visit the doctor? (*physical capability*)      1. Could you tell me what you had to do before you get an appointment to see the doctor for your diagnosis? (*physical and psychological capability*)      2. Did you require help to make an appointment? How was that like? (*physical capability, physical and social opportunity*)      3. What were the challenges you have encountered when visiting the doctor? (*physical and psychological capability*)      4. What would be helpful to you and your family to overcome these challenges? (*physical and psychological capability*)   3. Are you or your family likely to remember the date of the doctor’s appointment? (*psychological capability*)      1. Do you need anyone or anything to remind you of your doctor’s appointment? (*physical and social opportunity*)      2. Do you intend to continue seeing the doctor for your dementia/MCI diagnosis? It is okay to say no if that’s how you feel about it. (*reflective motivation*)   4. Is there anything preventing you from seeing the doctor? (*physical and social opportunity*) Please tell me more.   5. Were there times when you did not want to see the doctor or missed the doctor’s appointment? What happened? (*physical and social opportunity*)   **If not seeing the doctor**   - 1. What is holding you back from seeing a doctor? (*physical and social opportunity*)      1. What would help you to see a doctor for your diagnosis? (*reflective motivation*)      2. Do you need to see a doctor for any other health conditions? E.g., diabetes, high blood pressure, high cholesterol. (*physical and social opportunities*)      3. Could you share with me how come you are reluctant to see the doctor for your dementia/MCI condition? (*automatic and reflective motivation*)   2. If you were to see the doctor in the hospital to find out more about your dementia/MCI diagnosis, how would you do it? (*physical and psychological capability*)      1. How would you go about to make a doctor’s appointment in the polyclinic/clinic? (*physical and psychological capability*)      2. How would you get an (a referral) appointment to see the doctor in the hospital? (*physical and psychological capability*)      3. Would you be able to do it or your own? What kind of support do you require? (*physical and psychological capability*)   3. what do you think would happen to you if you don’t see a doctor for your dementia/MCI? (*reflective motivation*)      1. What are the consequences?      2. What are the benefits? |
| 1. What would be helpful to you and your family to cope and live with this diagnosis? E.g., caregiving support, financial support, know more information about dementia/MCI, community support. (*physical and social opportunity*)    1. At what point would these be useful? (*physical opportunity*)    2. How would you get these information/support? (*physical and social opportunity*) |
| **Transition to period when individual/family first noticed memory problem**  **For the next part of the interview, I would like to invite you to think back to the period when you or your family noticed you have memory problem. This is *before* you received the dementia/MCI diagnosis.** |
| 1. Before receiving the dementia/MCI diagnosis through PENSIEVE study, did you or anyone else notice you have any memory problem? (*psychological capability*)   **If yes**   - 1. What did you make of these memory problems? (*automatic motivation)*      1. How did you feel about the memory problems? (*automatic motivation*)      2. Did you take any actions to cope with the symptoms and/or feelings? (*automatic, reflective motivation*)   2. Did you or your family have any concerns that the memory problems could be serious? Please tell me more. (*reflective motivation*)      1. What were those concerns?      2. What did you do?   3. Have you or your family spoken about the memory problem to anyone outside your family? E.g., doctor, friend, social worker. (*social opportunity*)      1. Who did you speak to?      2. What made you decide to speak to that person?      3. What happened after that?   **If no**   1. Did your family or friends mention anything to you about their concern of your memory problem? **(If the question is directed at the caregiver, rephrase it to “Did your loved one mention any concerns about his/her memory?”)** (*social opportunity*)    1. If yes –       - 1. What were those concerns? (*reflective motivation*)         2. What did you do? (*reflective motivation*)    2. If no –       1. How would you feel if your family or friends mentioned to you about their concerns of your memory problem? (*automatic motivation*)       2. What would you have done if you heard concerns about your memory from your family or friends? **(If the question is directed at the caregiver, rephrase it to “How would you feel if your loved one mentioned concern about his/her memory to you?**       3. **What would you have done if you heard your loved one’s concern about his/her memory?”)** (*reflective motivation*) |
